# Tetravalent SARS-CoV-2 S1 subunit protein vaccination elicits robust humoral and cellular immune responses in SIV-infected rhesus macaque controllers

**DOI:** 10.1128/mbio.02070-23

**Published:** 2023-10-13

**Authors:** Muhammad S. Khan, Eun Kim, Quentin Le Hingrat, Adam Kleinman, Alessandro Ferrari, Jose C. Sammartino, Elena Percivalle, Cuiling Xu, Shaohua Huang, Thomas W. Kenniston, Irene Cassaniti, Fausto Baldanti, Ivona Pandrea, Andrea Gambotto, Cristian Apetrei

**Affiliations:** 1 Department of Surgery, University of Pittsburgh School of Medicine, Pittsburgh, Pennsylvania, USA; 2 Department of Infectious Diseases and Microbiology, University of Pittsburgh School of Public Health, Pittsburgh, Pennsylvania, USA; 3 Division of Infectious Diseases, Department of Medicine, University of Pittsburgh School of Medicine, Pittsburgh, Pennsylvania, USA; 4 Molecular Virology Unit, Microbiology and Virology Department, IRCCS Policlinico San Matteo, Pavia, Italy; 5 Department of Clinical, Surgical, Diagnostic and Pediatric Sciences, University of Pavia, Pavia, Italy; 6 Department of Pathology, University of Pittsburgh School of Medicine, Pittsburgh, Pennsylvania, USA; 7 UPMC Hillman Cancer Center, Pittsburgh, Pennsylvania, USA; University of California, Davis, California, USA

**Keywords:** COVID-19, vaccines, protein subunit, tetravalent, SARS-CoV-2, nonhuman primate, immunogenicity, efficacy, humoral immunity, cellular immunity

## Abstract

The coronavirus disease 2019 (COVID-19) pandemic has highlighted the need for safe and effective vaccines to be rapidly developed and distributed worldwide, especially considering the emergence of new severe acute respiratory syndrome coronavirus 2 (SARS-CoV-2) variants. Protein subunit vaccines have emerged as a promising approach due to their proven safety record and ability to elicit robust immune responses. In this study, we evaluated the immunogenicity and efficacy of an adjuvanted tetravalent S1 subunit protein COVID-19 vaccine candidate composed of the Wuhan, B.1.1.7 variant, B.1.351 variant, and P.1 variant spike proteins in a nonhuman primate model with controlled SIVsab infection. The vaccine candidate induced both humoral and cellular immune responses, with T and B cell responses mainly peaking post boost immunization. The vaccine also elicited neutralizing and cross-reactive antibodies, angiotensin-converting enzyme 2 (ACE2)-blocking antibodies, and T cell responses, including spike-specific CD4^+^ T cells. Importantly, the vaccine candidate was able to generate Omicron variant spike-binding and ACE2-blocking antibodies without specifically vaccinating with Omicron, suggesting potential broad protection against emerging variants. The tetravalent composition of the vaccine candidate has significant implications for COVID-19 vaccine development and implementation, providing broad antibody responses against numerous SARS-CoV-2 variants.

**IMPORTANCE:**

The study provides important insights into the immunogenicity and efficacy of a tetravalent protein subunit vaccine candidate against severe acute respiratory syndrome coronavirus 2 (SARS-CoV-2). The vaccine induced both humoral and cellular immune responses in nonhuman primates with controlled SIVagm infection and was able to generate Omicron variant-specific antibodies without specifically vaccinating with Omicron. These findings suggest that the tetravalent composition of the vaccine candidate could provide broad protection against multiple SARS-CoV-2 variants while minimizing the risk of immune escape and the emergence of new variants. Additionally, the use of rhesus macaques with controlled SIVsab infection may better represent vaccine immunogenicity in humans with chronic viral diseases, highlighting the importance of preclinical animal models in vaccine development. Overall, the study provides valuable information for the development and implementation of coronavirus disease 2019 vaccines, particularly for achieving global vaccine equity and addressing emerging variants.

## INTRODUCTION

The coronavirus disease 2019 (COVID-19) pandemic caused by the severe acute respiratory syndrome coronavirus 2 (SARS-CoV-2) has had an unprecedented impact on global health, the economy, and society. The COVID-19 pandemic consisted of over 675 million cases, with 6.5 million deaths, and 13 billion COVID-19 vaccine doses administered across the human population as of 3 February 2023 (1). Although approved COVID-19 vaccines have been effective in reducing mortality and morbidity caused by SARS-CoV-2 infection, the emergence of new variants that are able to evade the immune response has raised concerns about their long-term efficacy. Furthermore, the uneven distribution of vaccines worldwide has resulted in many low- to middle-income countries being left without access to variant-specific vaccines that are better suited for the evolving SARS-CoV-2 variant landscape. This highlights the need for the development of vaccines that can provide broad protection against a range of SARS-CoV-2 variants, as well as the importance of equitable distribution of vaccines to mitigate the risk of further virus evolution and spread (2 – 5). Since its emergence in late 2019, SARS-CoV-2 has continuously evolved at a higher-than-expected rate, giving rise to multiple variants with multiple genetic mutations and various phenotypic properties, including increased transmissibility, virulence, and immune escape (5, 6). The emergence of these variants has raised concerns about the efficacy of current vaccines and the potential for future outbreaks. Therefore, there is a critical need to develop effective vaccines that can provide broad and durable protection against SARS-CoV-2 and its variants. SARS-CoV-2 variants such as B.1.1.7 (Alpha), B.1.351 (Beta), and P.1 (Gamma) have exhibited substantial ability to escape immune responses induced by wild-type (WU) vaccines or natural infections (7, 8).

The spike (S) protein of SARS-CoV-2 has been the main target of currently approved COVID-19 vaccines and of most COVID-19 vaccines in development (9). S protein allows for virus binding and infection of susceptible cells through interaction with its host receptor, angiotensin-converting enzyme 2 (ACE2) (10). The S1 subunit of the S protein contains the receptor binding domain (RBD) that binds to ACE2, while the S2 subunit allows for cell fusion and viral entry (11, 12). It has been widely acknowledged that antibodies targeting the S protein, particularly those binding to the RBD, are able to block SARS-CoV-2 binding to the cell receptor and prevent infection of susceptible cells (13 – 17). We have previously demonstrated the immunogenicity of S1 subunit-targeting vaccines against various beta-coronaviruses, including SARS-CoV-1, SARS-CoV-2, and Middle East respiratory syndrome (MERS) (18 – 23). Particularly, our previous work with MERS comparing S1-based vaccines to whole S vaccines demonstrated that S1-based vaccines induce greater neutralizing antibody responses than whole S vaccines (19), which was the rationale for employing only S1 in our study.

A focus for next-generation SARS-CoV-2 vaccine design is the investigation of novel vaccines that may be able to induce a broader immune response effective against multiple SARS-CoV-2 variants. A multivalent vaccine is a traditional approach used to increase antigen coverage against ever-changing pathogens such as COVID-19, which has numerous variants, some of them cocirculating (24 – 26). Multivalent vaccine approaches have been shown to increase the immunogenicity of various vaccines, especially in the context of influenza (27 – 31). We have previously demonstrated the immunogenicity of a trivalent protein subunit vaccine in BALB/c mice (22). We have evaluated the immunogenicity of unadjuvanted wild-type (WU S1-RS09cg) and variant-specific (Delta S1-RS09cg and OM S1-RS09cg) S1 subunit protein vaccines delivered either as a monovalent or a trivalent antigen in BALB/c mice (22). Our previous results show that a trivalent approach induced a broader humoral response with more coverage against antigenically distinct variants (22). The trivalent approach was also found to have increased or equivalent ACE2 binding inhibition and increased S1 IgG endpoint titer at early time points against SARS-CoV-2 spike variants when compared to monovalent Wuhan, Delta, or Omicron S1 (22). Here, we assessed our S1 protein subunit vaccine, at an increased valency to tetravalent, in an advanced animal model more closely related to humans. Nonhuman primates (NHPs) are commonly used as preclinical models to evaluate the safety and efficacy of vaccines and therapeutics for infectious diseases, including SARS-CoV-2 (32 – 35). We employed a rhesus macaque (RM) model of controlled simian immunodeficiency virus (SIV) infection to evaluate the immunogenicity of a tetravalent SARS-CoV-2-S1 protein subunit vaccine delivered with AddaVax adjuvant. Controlled SIV infection in RMs mimics a situation of chronic viral infection that can be encountered in humans, which may influence the development of immune responses to vaccination. Indeed, some studies reported lower SARS-CoV-2 antibody responses for people living with HIV (36, 37). Several studies have demonstrated the utility of RMs as a preclinical model for SARS-CoV-2 vaccine development. For example, macaques have been used to evaluate the immunogenicity and correlates of protection, as well as the protective efficacy of various vaccine platforms, including viral vector-based vaccines, mRNA vaccines, and protein subunit vaccines (34, 35, 38 – 42). Moreover, the use of NHP models can provide critical insights into the mechanisms of vaccine-induced immunity, including the kinetics, specificity, and durability of the immune responses. In this study, we utilized an RM model with controlled SIVsab infection (43) in the absence of antiretroviral therapy through an effective cellular immune response (44). In this model, a robust acute SIV infection with high levels of viral replication and dramatic mucosal CD4^+^ T cell depletion, similar to pathogenic HIV-1/SIV infections of humans and RMs, is followed by a complete and durable control of virus replication, defined as undetectable viral loads (VLs) in blood and tissues beginning 72 to 90 days postinoculation and continuing at least 4 years; seroreversion; progressive recovery of mucosal CD4^+^ T cells, with complete recovery by 4 years postinoculation; normal levels of T cell immune activation, proliferation, and apoptosis; and no disease progression (43). This “functional cure” of SIVsab infection can be reverted after multiple years of control by depleting CD8^+^ cells, resulting in transient rebounds of viral loads that are again controlled with the restoration of CD8^+^ cells (43).

Here, we evaluated the immunogenicity of a tetravalent SARS-CoV-2 vaccine approach with an S1 subunit protein vaccine targeting Wuhan S1, B.1.1.7 (Alpha), B.1.351 (Beta), and P.1 (Gamma). We chose these variants because, at the time of the start of the study, they represented a diverse and relevant set of SARS-CoV-2 strains that circulated in different regions of the world and had distinct mutations in the spike protein, which is the main target of neutralizing antibodies. We found that vaccination induced robust humoral and cellular immune responses, which resulted in antibodies capable of blocking ACE2 binding to 15 different SARS-CoV-2 variants, including multiple Omicron variants. Vaccination also induced antibodies that were able to block SARS-CoV-2 infection of susceptible cells by live wild-type (WU), Beta, and Delta variant viruses. We profiled the lymphocyte response to immunization for 2 months post initial prime vaccination by quantifying the number of T and B cells, investigating markers of T cell activation, and investigating memory subsets in peripheral blood mononuclear cells (PBMCs) and showed robust immune activation, primarily after boost immunization. We were also able to measure a spike-specific CD4^+^ T cell response in the PBMCs of RMs 42 days post prime immunization, although no CD8^+^ T cell response was found. Our study further demonstrates the immunogenicity of protein subunit vaccines against SARS-CoV-2 targeting the S1 subunit of the spike protein while also contributing insights on approaches to further increase the valency of currently approved COVID-19 vaccines.

## RESULTS

### Design and expression of recombinant proteins

**FIG 1:**
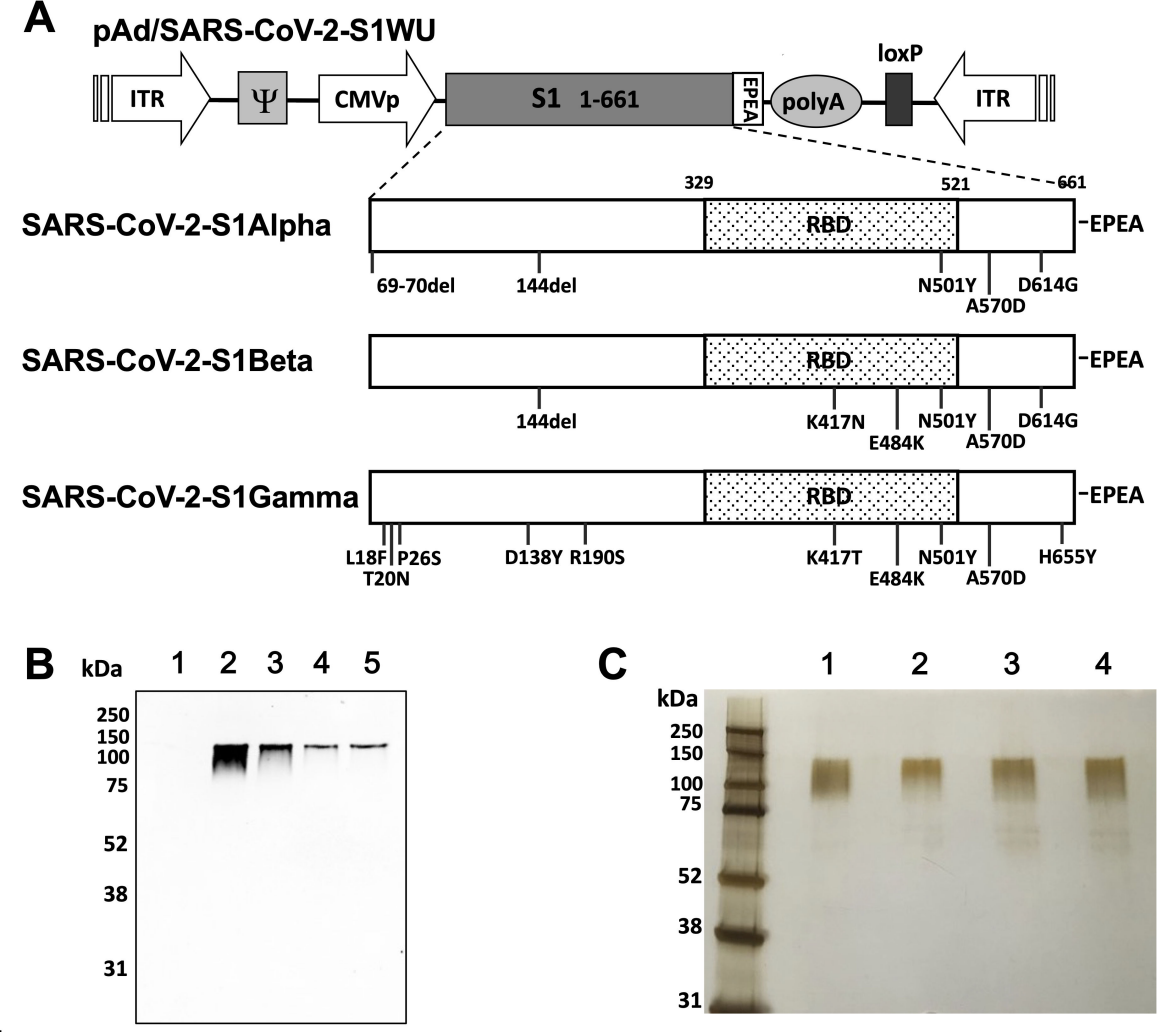
Construction and expression of tetravalent recombinant SARS-CoV-2-S1 proteins. (A) A shuttle vector carrying the codon-optimized four variants of the SARS-CoV-2-S1 gene encoding N-terminal 1–661 with a c-tag (EPEA) was designated as shown in the diagram. Amino acid changes in the SARS-CoV-2-S1 region of this study are shown. ITR, inverted terminal repeat; RBD, receptor binding domain. (**B)** Detection of the SARS-CoV-2-S1 proteins by western blot with the supernatant of Expi293 cells transfected with pAd/S1WU (lane 2), pAd/S1Alpha (lane 3), pAd/S1Beta (lane 4), and pAd/S1Gamma (lane 5), respectively, using rabbit anti-SARS-CoV Wuhan polyclonal antibody. As a negative control, mock-transfected cells were treated the same (lane 1). (**C**) Purified proteins, rS1WU (lane 1), rS1Alpha (lane 2), rS1Beta (lane 3), and rS1Gamma (lane 4), isolated by c-tag affinity purification, were separated by SDS-PAGE and visualized by silver staining. A molecular weight marker (MW marker) is indicated on the left.

To produce recombinant proteins of SARS-CoV-2-S1, pAd/S1Wu, pAd/S1Alpha, pAd/S1Beta, and pAd/S1Gamma were generated by subcloning the codon-optimized SARS-CoV-2-S1 gene having a C-tag into the shuttle vector pAd (GenBank number U62024) at *Sal* I and *Not* I sites (Fig. 1A). Variant-specific mutations for B.1.1.7 (Alpha), B.1.351 (Beta), and P.1 (Gamma) SARS-CoV-2 recombinant S1 proteins are outlined. To determine SARS-CoV-2-S1 expression from each plasmid, Expi293 cells were transfected with pAd/S1WU, pAd/S1Alpha, pAd/S1Beta, and pAd/S1Gamma, or pAd as a control. At 5 days after transfection, the supernatants of Expi293 cells were characterized by western blot analysis. As shown in Fig. 1B, each S1 recombinant protein was recognized by a polyclonal anti-spike of SARS-CoV-2 Wuhan antibody at the expected glycosylated monomeric molecular weights of about 110 kDa under denaturing-reduced conditions, while no expression was detected in the mock-transfected cells (lane 1). The purified rS1WU, rS1Apha, rS1Beta, and rS1Gamma proteins using the C-tagXL affinity matrix were determined by silver staining (Fig. 1C).

### Binding antibody and cross-variant live virus neutralizing antibody response

**FIG 2:**
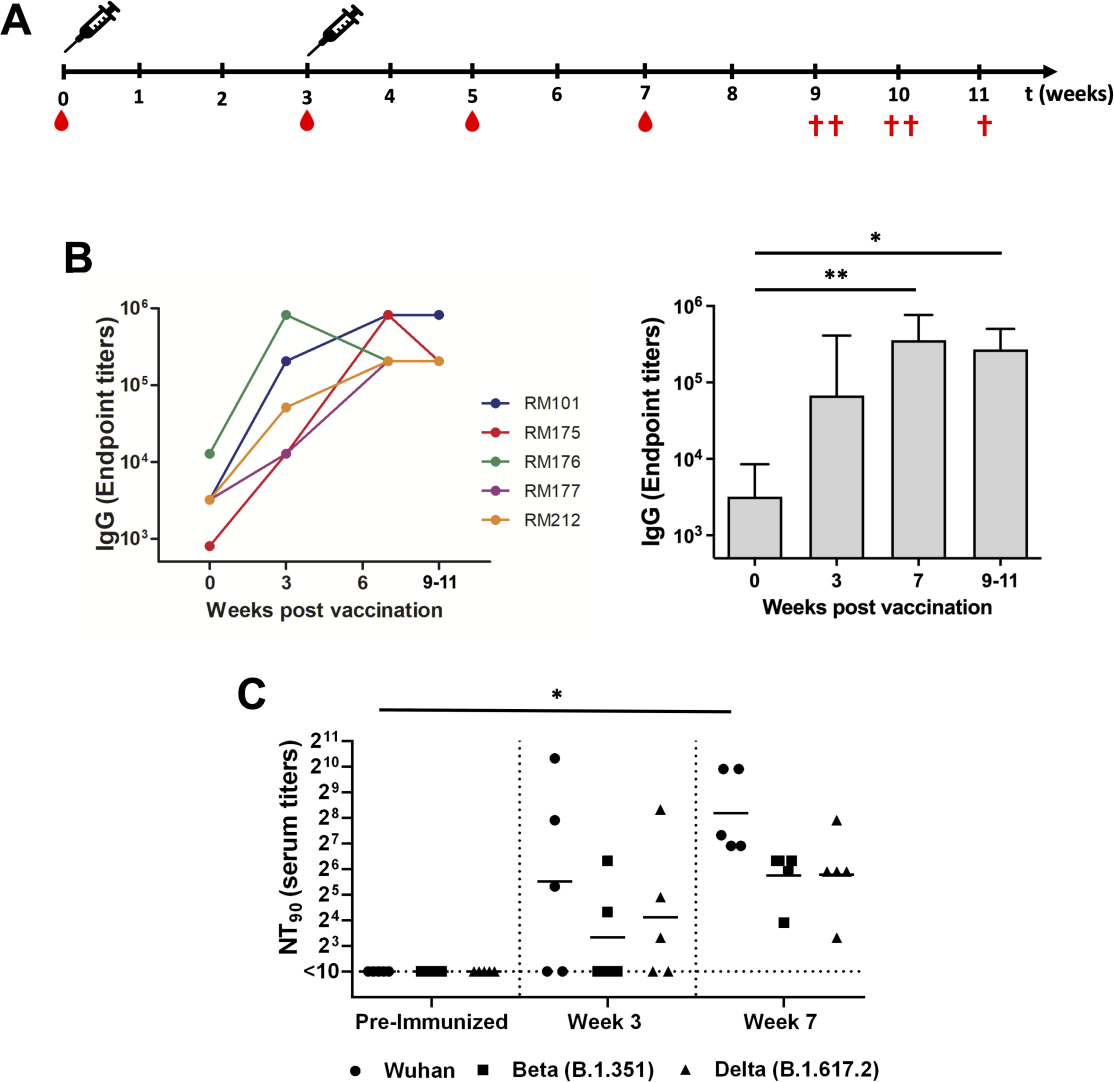
Antigen-specific antibody responses in rhesus macaques immunized with a tetravalent SARS-CoV-2 rS1 protein subunit vaccine. (A) Schedule of immunization and blood sampling for IgG endpoint titration. Rhesus macaques (*N* = 5) were immunized with 60 µg of tetravalent rS1 proteins of Wuhan, B.1.1.7 (Alpha), B.1.351 (Beta), and P.1 (Gamma) (15 µg of each antigen) mixed with AddaVax adjuvant, then administered to the RMs arm at weeks 0 and 3. Syringes indicated the timing of immunization, and the red drops denote the times at which blood was drawn. The red crosses showed the euthanized times of each RM. (**B**) Sera were diluted, and SARS-CoV-2-S1-specific antibodies were quantified by ELISA to determine the IgG endpoint titer. The IgG titers at each time point were shown in each RM. The bars represent the geometric mean with a geometric SD. (**C**) Neutralizing antibodies in the serum of RMs prior to immunization, along with weeks 3 and 7 post immunization, were measured using a microneutralization assay (NT_90_) with SARS-CoV-2 Wuhan, Beta, and Delta. Serum titers that resulted in a 90% reduction in cytopathic effect compared to the virus control were reported. Horizontal lines represent geometric mean titers. Groups were compared by the Kruskal-Wallis test at each time point, followed by Dunn’s multiple comparison test. Significant differences are indicated by *P < 0.05, **P < 0.005. *N* = 5 rhesus macaques per group for each experiment.

Prior to immunization, RMs were infected with a simian immunodeficiency virus (SIV) that naturally infects African green monkeys (SIVsab) (45). This virus is completely controlled in RMs (44), in spite of retaining its replicative abilities (43). At the time of SARS-CoV-2 immunization, the RMs had been controlling SIVsab for over a year. Upon prime and boost immunization, SIVsab viral loads remained undetectable throughout the follow-up, suggesting no SIV activation upon vaccination. RMs were primed and boosted on week 3 with 60 µg total of rS1WU, rS1Apha, rS1Beta, and rS1Gamma, 15 µg of each antigen, mixed with 300 µL of AddaVax, a squalene-based oil in water nano-emulsion adjuvant (Fig. 2A). To assess the magnitude of the antibody response, we first determined Wuhan IgG antibody endpoint titers in the sera of vaccinated RMs with enzyme linked immunosorbent assay (ELISA). Serum samples collected prior to immunization and then at weeks 3, 7, and 9–11 after immunization were serially diluted to determine SARS-CoV-2-S1-specific IgG titers against Wuhan S1 using ELISA (Fig. 2B). RMs had detectable anti-S1 binding antibody responses prior to immunization (Fig. 2B); however, no neutralizing antibody response was found (Fig. 2C). S1-specific IgG titers were statistically increased at weeks 7 and 9–11 when compared to week 0 (Fig. 2B, *P* < 0.05, Kruskal-Wallis test, followed by Dunn’s multiple comparison test). To evaluate the functional quality of vaccine-generated antigen-specific antibodies, we used a microneutralization assay (NT_90_) to test the ability of sera from immunized RMs to neutralize the infectivity of SARS-CoV-2. Sera collected from RMs on weeks 3 (prior to booster immunization) and 7 (4 weeks post boost) after primary immunization were tested for the presence of SARS-CoV-2-specific neutralizing antibodies with live SARS-CoV-2 Wuhan, Beta, and Delta viruses (Fig. 2C). High levels of neutralizing antibodies were detected in sera at weeks 3 and 7 against Wuhan, Beta, and Delta SARS-CoV-2 variants (Fig. 2C) and showed a similar pattern with IgG endpoint titers in each RM (Fig. S2). Furthermore, the geometric mean titers (GMTs) of neutralizing antibodies at week 7 against the Wuhan, Beta, and Delta strains were increased 6.4-, 5.4-, and 3.2-folds compared to week 3, respectively, while only the neutralizing antibody response against live Wuhan SARS-CoV-2 at week 7 was significantly increased when compared to preimmunized sera (Fig. 2C, *P* < 0.05, Kruskal-Wallis test, followed by Dunn’s multiple comparison test). Neutralization against highly immune-evasive Beta and Delta SARS-CoV-2 variants of concern (VOC) was found to be slightly lower than that induced by the Wuhan variant at both weeks 3 and 7 (Fig. 2C). While Beta VOC S1 was included in the tetravalent immunization regimen, Delta VOC was not, highlighting the diverse response induced by tetravalent immunization in RMs.

### A tetravalent vaccine induces potent ACE2 binding inhibition effective against 15 different SARS-CoV-2 VOC spikes

**FIG 3:**
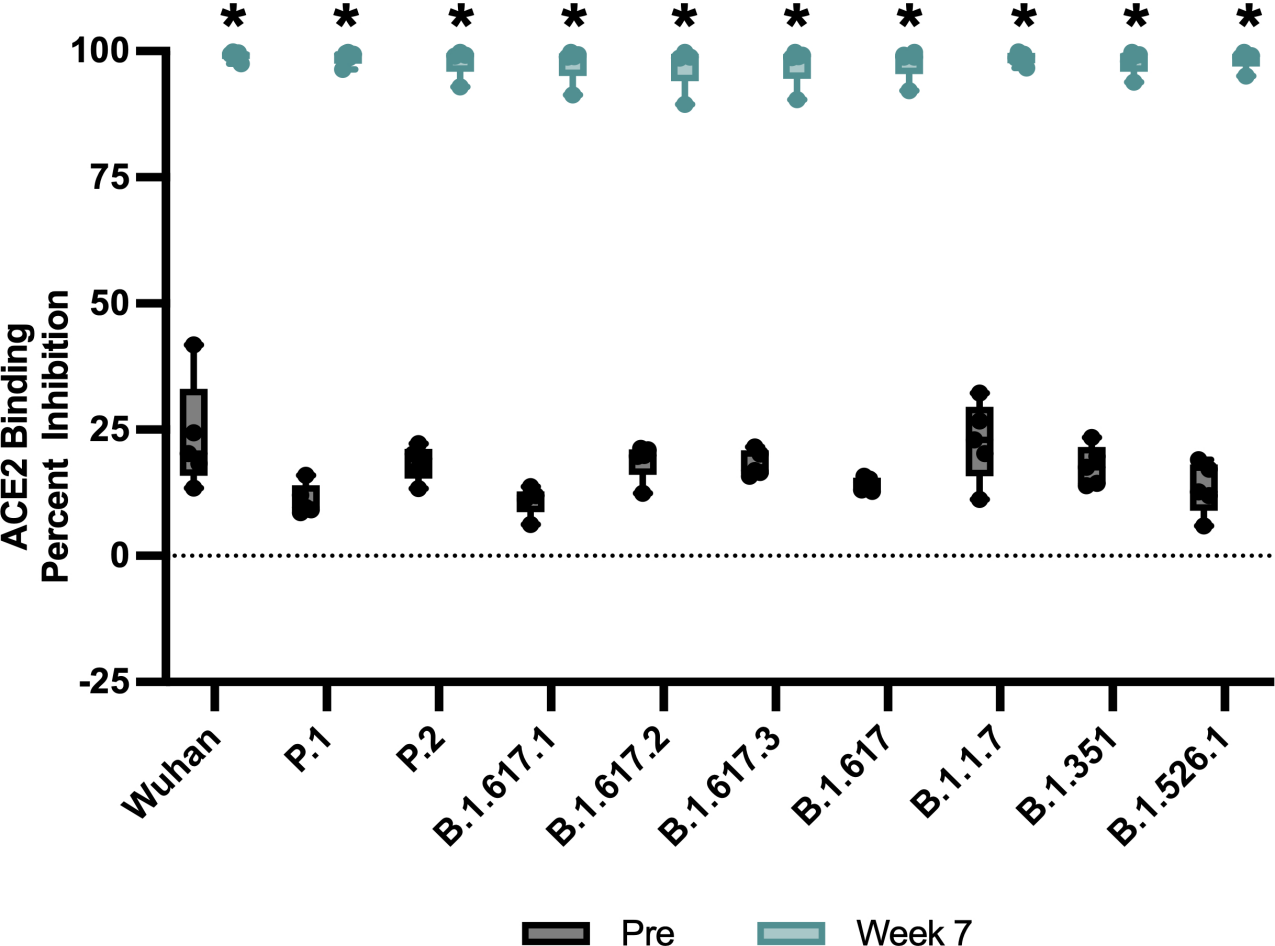
Percent ACE2 binding inhibition of neutralizing antibodies against SARS-CoV-2 variants. Antibodies in sera (diluted 1:10) capable of neutralizing the interaction between SARS-CoV-2 Wuhan, Alpha (B.1.1.7), Beta (B.1.351), Gamma (P.1), Delta (B.1.617.2), Zeta (P.2), Kappa (B.1.617.1), New York (B.1.516.1), and India (B.1.617 and B.1.617.3) variant spikes and ACE2 were examined in all animals’ preimmunization and week 7 post prime immunization with V-PLEX SARS-CoV-2 Panel 18. Groups were compared by Kruskal-Wallis test at each time point to a preimmunized serum control, followed by Dunn’s multiple comparison test Significant differences are indicated by *P < 0.05. *N* = 5 rhesus macaques per group for each experiment.

For further insight into the neutralizing capabilities of antibodies induced by vaccination, we used the Meso Scale Discovery (MSD) V-PLEX SARS-CoV-2 (ACE2) Kit to measure the inhibition of binding between ACE2 and the trimeric spike protein of SARS CoV-2 variants. Initially, we used kit Panel 18, including Wuhan S and spikes from variants Alpha (B.1.1.7), Beta (B.1.351), Gamma (P.1), Delta (B.1.617, B.1.617.2), Zeta (P.2), Kappa (B.1.617.1), B.1.526.1, B.1.617, and B.1.617.3 (Fig. 3). Sera from vaccinated RMs were examined at week 7, due to that being the peak of the measured IgG-binding antibody response, and compared to preimmunized sera (Fig. 2A; Fig. 3). Antibodies blocking ACE2 and trimeric S binding of all variants by over 90% inhibition were detected in all 1:10 diluted RM sera at week 7 (Fig. 3). Week 7 sera ACE2 binding inhibition for RMs was significantly increased, when compared to preimmunized sera, for Wuhan, B.1.1.7, B.1.351, P.1, B.1.617.2, P.2, B.1.617.1, B.1.526.1, B.1.617, and B.1.617.3 spike (Fig. 3, *P* < 0.05, Mann-Whitney test).

**FIG 4:**
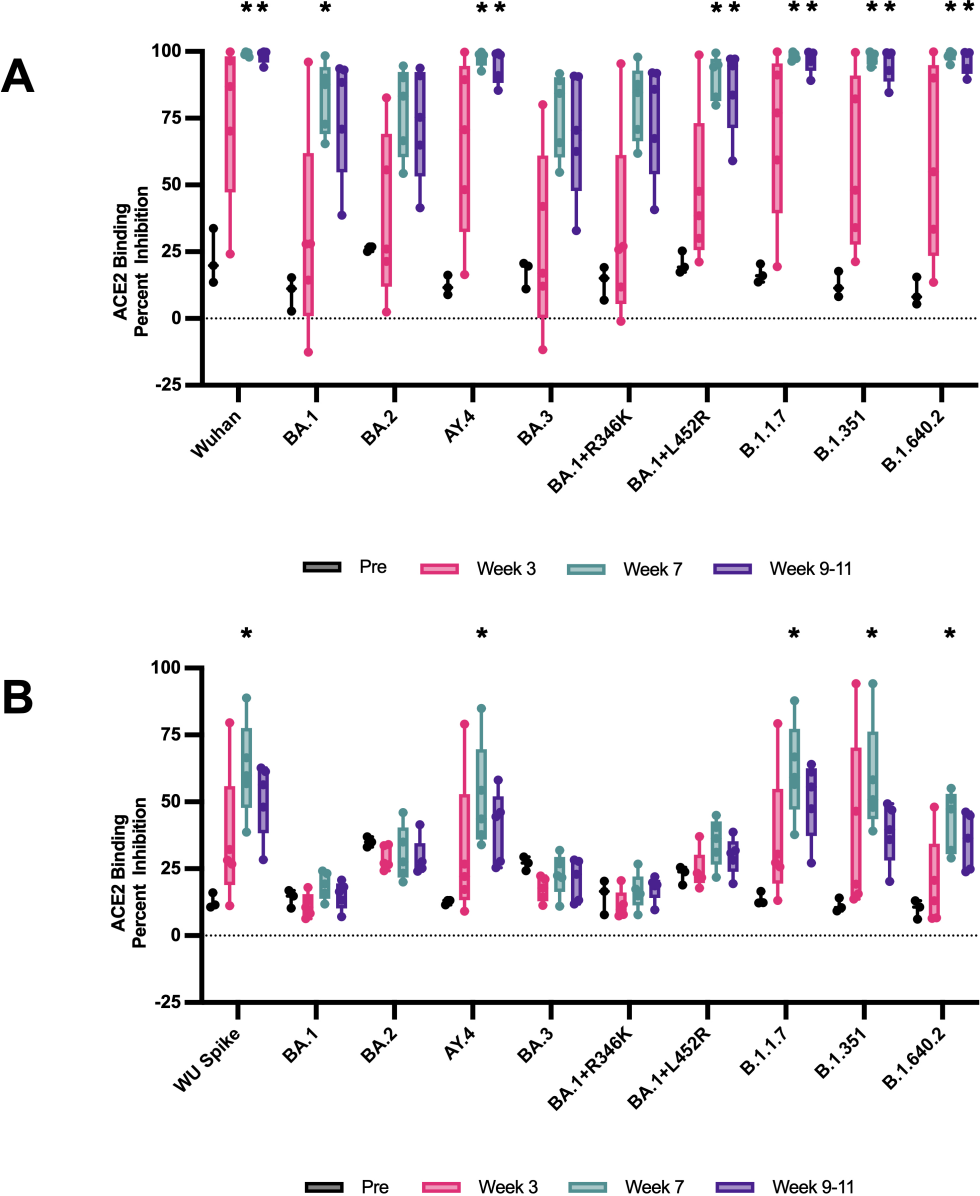
Percent ACE2 binding inhibition of neutralizing antibodies against Omicron SARS-CoV-2 variants. Antibodies in sera, diluted (**A) **1:10 and (**B**) 1:100, capable of blocking the binding of SARS-CoV-2 spikes, including Wuhan and spikes from immune evasive variants (BA.1, BA.2, AY.4 [Delta lineage], BA.3, BA.1 + R346K mutation, BA.1 + L452R mutation, B.1.1.7 [Alpha], B.1.351 [Beta], and B.1.1640.2), to ACE2 were detected with a V-PLEX SARS-CoV-2 Panel 25. Groups were compared by Kruskal-Wallis test at each time point to a preimmunized serum control, followed by Dunn’s multiple comparison test. Significant differences are indicated by *P < 0.05. *N* = 5 rhesus macaques per group for each experiment.

To assess the neutralizing capabilities of RM vaccine-induced antibodies against Omicron (BA.1) VOC and Omicron sub-variants (BA.2, BA.3, BA.1 + R346K, BA.1 + L452R), we used MSD V-Plex SARS-CoV-2 ACE2 Kit Panel 25 (Fig. 4). Panel 25 includes SARS-CoV-2 Wuhan, BA.1, BA.2, AY.4, BA.3, BA.1 + R346K, BA.1 + L452, B.1.1.7, B.1.351, and B.1.640.2 trimeric spikes. Sera from vaccinated RMs were examined at weeks 3, 7, and 9–11 postvaccination and compared to preimmunized sera at 1:10 dilution (Fig. 4A) and 1:100 dilution (Fig. 4B). Weeks 7 and 9–11 RM sera’s ACE2-binding inhibition were significantly increased when compared to preimmunized sera for Wuhan, AY.4 (Delta lineage), BA.1 + L452R, B.1.1.7, B.1.351, and B.1.640.2 VOC spikes at 1:10 dilution (Fig. 4A, *P* < 0.05, Kruskal-Wallis test, followed by Dunn’s multiple comparison test). Week 7 RM sera’s ACE2-binding inhibition were significantly increased when compared to preimmunized sera for BA.1 VOC spike at 1:10 dilution (Fig. 4A, *P* < 0.05, Kruskal-Wallis test, followed by Dunn’s multiple comparison test). While not statistically significantly increased when compared to preimmunized RM sera, RMs demonstrated moderate ACE2-binding inhibition for BA.2, BA.3, and BA.1 + R346K VOC spikes weeks 7 and 9–11 post immunization at 1:10 dilution (Fig. 4A, *P* > 0.05, Kruskal-Wallis test, followed by Dunn’s multiple comparison test). To further interrogate the vaccine-induced neutralizing capabilities of RMs, we substantially diluted RM sera to 1:100 (Fig. 4B). Week 7 RM 1:100 diluted sera’s ACE2 binding inhibition were significantly increased when compared to preimmunized sera for Wuhan, AY.4, B.1.1.7, B.1.351, and B.1.640.2 VOC spikes (Fig. 4B, *P* < 0.05, Kruskal-Wallis test, followed by Dunn’s multiple comparison test). At 1:100 dilution, RM sera did not have ACE2 binding inhibition above preimmunized sera for BA.1, BA.2, BA.3, BA.1 + R346K, and BA.1 + L452R VOC spikes (Fig. 4B). Results suggest the necessity of booster immunization to induce potent and cross-variant-recognizing antibodies. Results also suggest that vaccination-induced antibodies that are able to potently recognize and block ACE2 binding of a wide range of SARS-CoV-2 variant spikes by week 7 post prime immunization.

### Longitudinal lymphocyte dynamics and cell-mediated immune response to vaccination show that immune activation is primarily observed after a boost

To investigate the kinetics and magnitude of immune responses induced by the tetravalent SARS-CoV-2 vaccine, we monitored the PBMCs of vaccinated rhesus macaques over a 60-day period. PBMCs are a mixture of different immune cell types, including T and B cells, and are a useful tool for investigating the immune response to vaccination *in vivo*.

**FIG 5:**
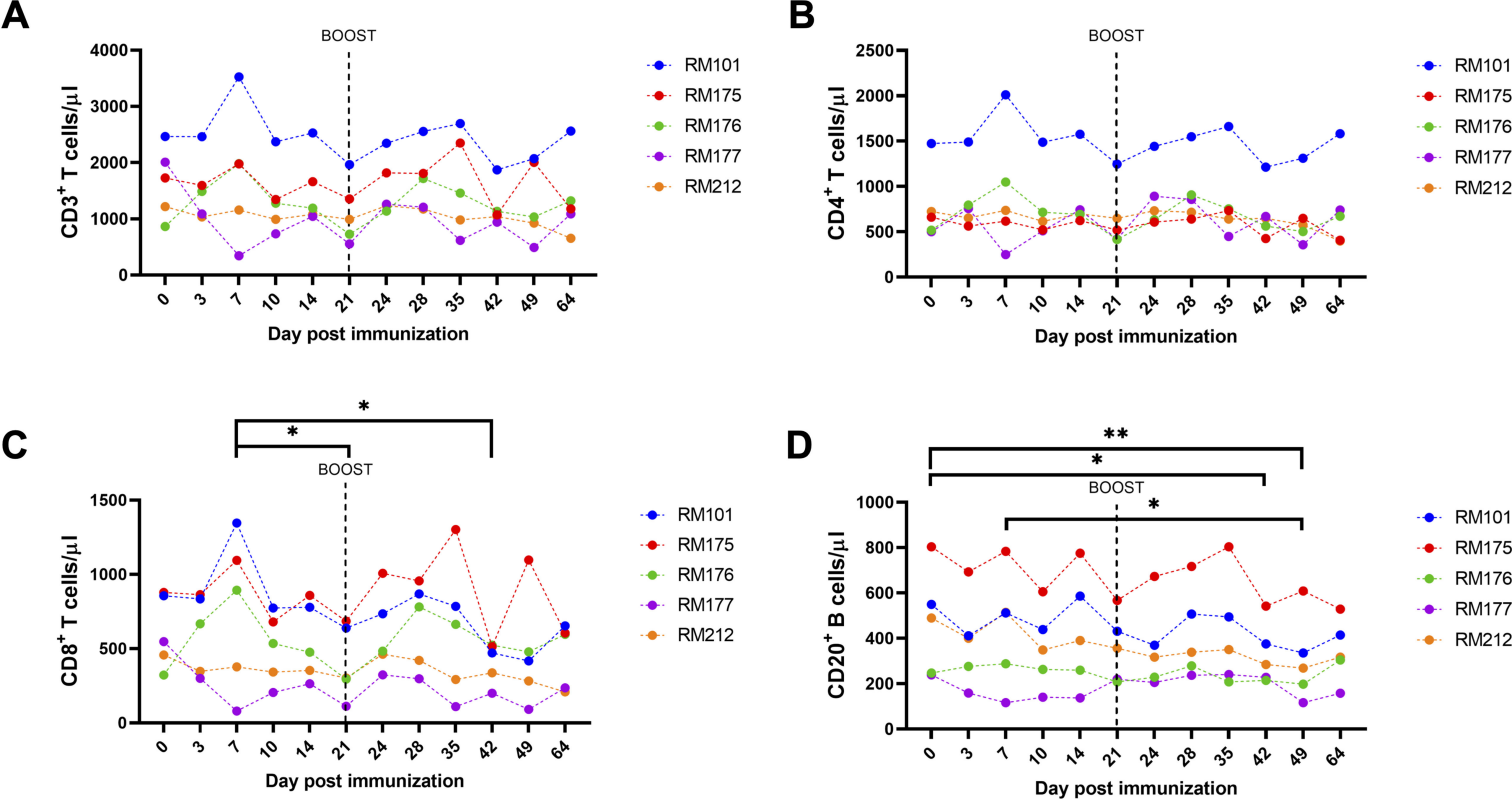
CD3, CD4, CD8, and CD20 cell counts post immunization and boost. Absolute counts of immune cells in whole blood and immunophenotyping of circulating immune cells were determined by flow cytometry. Then, 50 µL of whole blood was added to a TruCount tube (BD Biosciences) containing an antibody mix, allowing us to precisely quantify (**A) **CD45^+^ cells, (**B**) CD4^+^ cells, (**C**) CD8^+^ T cells, and (**D) **CD20^+^ B cells in blood per μL. PMBCs from RMs were collected and analyzed on days −1, 3, 7, 10, 14, 21, 24, 28, 31, 35, 42, 49, and 64 post prime immunization. Individual results for each RM are depicted. Groups were compared by two-way ANOVA, followed by Tukey’s multiple comparison test on the mean results at each time point.

Figure 5 shows the dynamics of CD3^+^ T cell (Fig. 5A), CD4^+^ T cell (Fig. 5B), CD8^+^ T cell (Fig. 5C), and CD20^+^ B cell (Fig. 5D) counts over 60 days. We observed increases in all T cell subsets (CD3^+^, CD4^+^, and CD8^+^) and B cells (CD20^+^) after the prime and after the boost immunization, demonstrating clear increases for all subsets. Mean CD8^+^ cell counts were significantly higher at day 7 post immunization than at days 21 and 42 [Fig. 5C, *P* < 0.05, two-way analysis of variance (ANOVA), followed by Tukey’s multiple comparison test]. Mean CD20^+^ B cell counts were significantly decreased at days 42, 49, and 64 when compared to day 0, indicating a reduced number of circulating memory B cells in the peripheral blood after immunization (Fig. 5D, *P* < 0.05, two-way ANOVA, followed by Tukey’s multiple comparison test).

**FIG 6:**
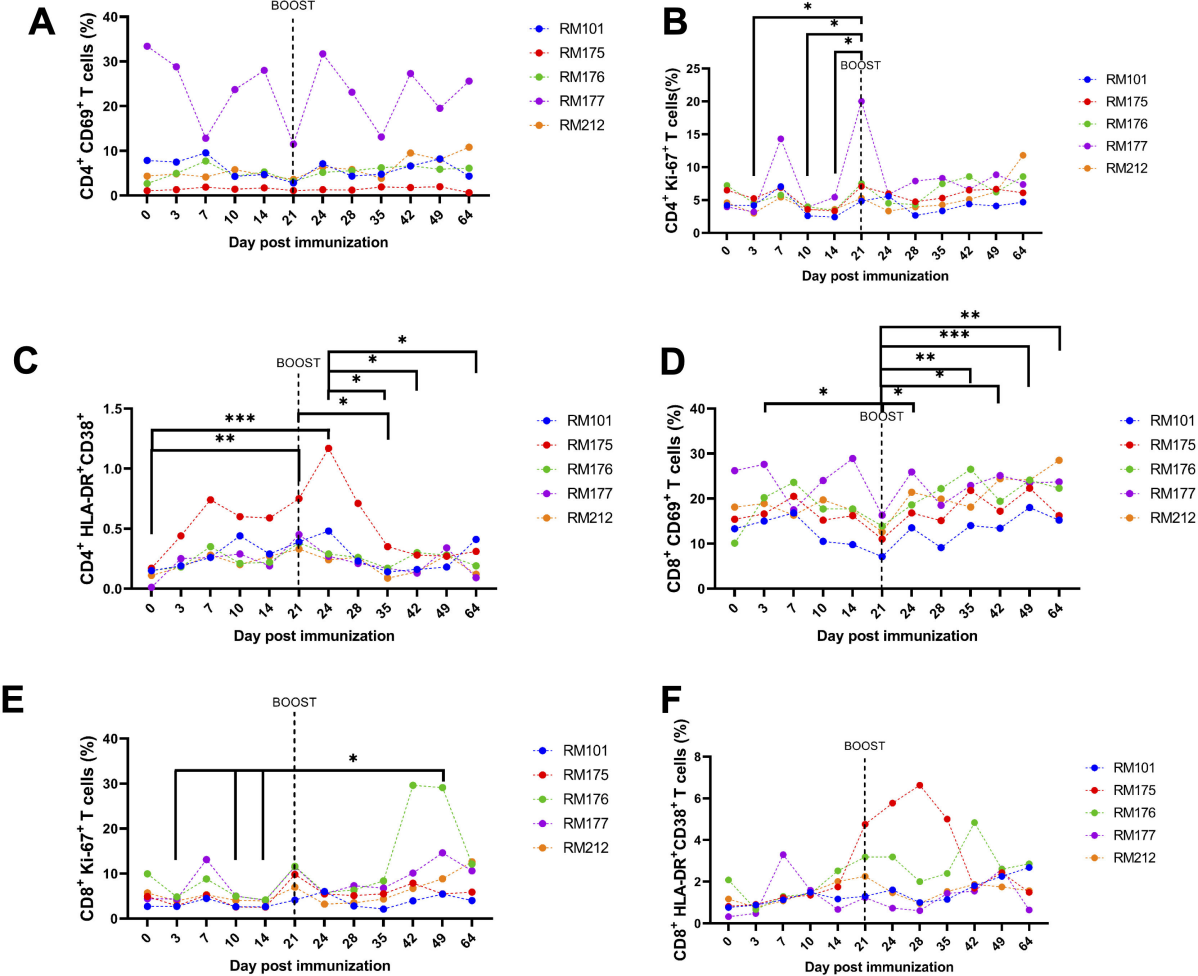
CD4 and CD8 T cell activation post immunization and boost. Whole peripheral blood was stained with fluorescently labeled antibodies for CD4^+^, CD8^+^, CD69^+^, Ki-67^+^, and HLA-DR^+^ to investigate CD4 and CD8 activation induced by vaccination with flow cytometry. (**A) **Frequencies of CD4^+^ CD69^+^ T cells. (**B**) Frequencies of CD4^+^ Ki-67^+^ T cells. (**C) **Frequencies of CD4^+^ HLA-DR^+^ CD38^+^ T cells. (**D) **Frequencies of CD8^+^ CD69^+^ T cells. (**E) **Frequencies of CD8^+^ Ki-67^+^ T cells. (**F) **Frequencies of CD8^+^ HLA-DR^+^ CD38^+^ T cells. PMBCs from RMs were collected and analyzed on days −1, 3, 7, 10, 14, 21, 24, 28, 31, 35, 42, 49, and 64 post prime immunization. Individual results for each RM are depicted. Groups were compared by two-way ANOVA, followed by Tukey’s multiple comparison test on the mean results at each time point.

Figure 6 shows the fraction of activating and proliferating CD4^+^ and CD8^+^ T cells. We used the activation markers CD69, HLDR, and CD38, as previously described in the literature (46 – 48). We also used Ki-67 as a marker for cell proliferation. CD69^+^ CD4^+^ T cell induction was mainly observed in RM177 (Fig. 6A). Ki67^+^ CD4^+^ T cells showed significant increases in mean percentage at day 21 post prime immunization, prior to boost vaccination, when compared to days 3, 10, and 14 (Fig. 6B, *P* < 0.05, two-way ANOVA, followed by Tukey’s multiple comparison test). HLA-DR^+^ CD38^+^ CD4^+^ T cells showed activation post prime and boost with a return to near baseline by day 40, with days 21 and 24 mean fractions being significantly increased when compared to day 0 (Fig. 6C, *P* < 0.05, two-way ANOVA, followed by Tukey’s multiple comparison test). Additionally, the mean percentage of HLA-DR^+^ CD38^+^ CD4^+^ T cells was significantly decreased at day 35 when compared to days 21 and 24, along with being significantly decreased at days 42 and 64 when compared to day 24 (Fig. 6C, *P* < 0.05, two-way ANOVA, followed by Tukey’s multiple comparison test). The fraction of CD69^+^ CD8^+^ T cells increased in all RMs post prime and boost, with most starting to return to prevaccination levels at day 60 (Fig. 6D). The mean fraction of CD69^+^ CD8^+^ T cells was significantly decreased at day 21 when compared to day 3 (Fig. 6D, *P* < 0.05, two-way ANOVA, followed by Tukey’s multiple comparison test). Additionally, the mean fraction of CD69^+^ CD8^+^ T cells was significantly increased at days 24, 35, 42, 48, and 64 when compared to day 21, showing the degree of activation after boost immunization (Fig. 6D, *P* < 0.05, two-way ANOVA, followed by Tukey’s multiple comparison test). The induction of Ki-67^+^ CD8^+^ T cells was primarily seen after day 40 post immunization (Fig. 6E), with mean fractions being significantly increased at day 49 when compared to days 3, 10, and 14 (Fig. 6E, *P* < 0.05, two-way ANOVA, followed by Tukey’s multiple comparison test). HLA-DR^+^ CD38^+^ CD8^+^ T cell activation was mainly seen in RM175 and RM176 at different time points (Fig. 6F). However, the induction of HLA-DR^+^ CD38^+^ CD8^+^ T cells was not as robust as that of CD69^+^ CD8^+^ T cells and Ki-67^+^ CD8^+^ T cells (Fig. 6F, D, and E).

**FIG 7:**
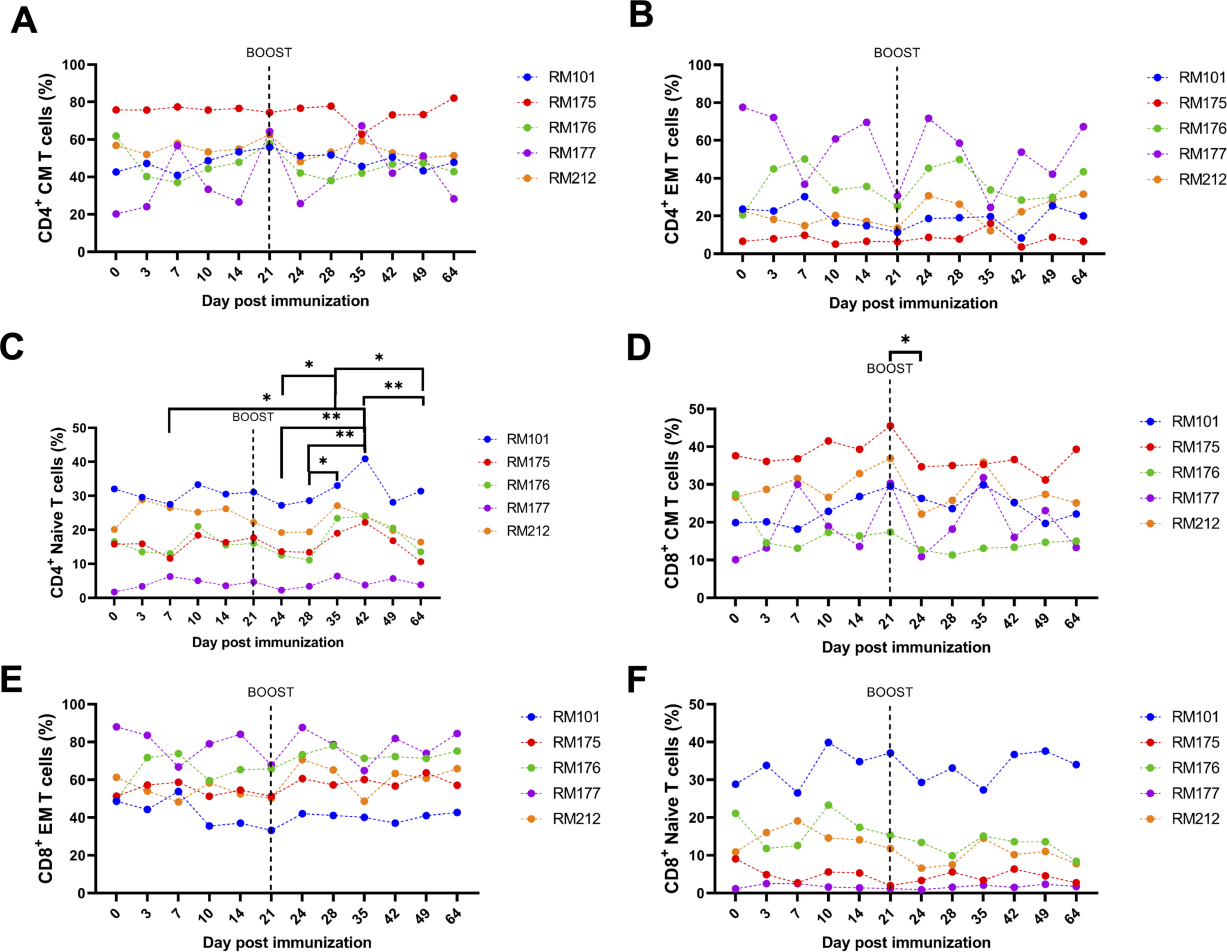
T cell memory subset dynamics and induction post immunization and boost. Whole peripheral blood was stained with fluorescently labeled antibodies for CD4^+^, CD8^+^, CD28^+^, and CD95^+^. Memory subsets were defined for naïve, CM, and EM T cells using CD28^+^ and CD95^+^ markers. Naïve T cells are CD28^+^CD95^−^; CM T cells are CD28^+^CD95^+^; and EM T cells are CD28-CD95^+^. (**A) **Frequencies of CD4^+^ CM T cells. (**B) **Frequencies of CD4^+^ EM T cells. (**C) **Frequencies of CD4^+^ naïve T cells. (**D) **Frequencies of CD8^+^ CM T cells. (**E) **Frequencies of CD8^+^ EM T cells. (**F) **Frequencies of CD8^+^ naïve T cells. PMBCs from RMs were collected and analyzed on days −1, 3, 7, 10, 14, 21, 24, 28, 31, 35, 42, 49, and 64 post prime immunization. Individual results for each RM are depicted. Groups were compared by two-way ANOVA, followed by Tukey’s multiple comparison test on the mean results at each time point.

Figure 7 shows the changes in the distribution of T cell memory subsets over time. We defined naïve, central memory (CM), and effector memory (EM) T cells using CD28^+^ and CD95^+^ markers. Naïve T cells are CD28^+^ CD95^neg^; CM T cells are CD28^+^ CD95^+^; and EM T cells are CD28^neg^ CD95^+^. We observed moderate decreases in abundance of CD4^+^ and CD8^+^ central memory T cells (Fig. 7A and D), along with naïve CD4^+^ and naïve CD8^+^ T cells (Fig. 7C and F), after prime and boost. CD4^+^ and CD8^+^ effector memory T cells (Fig. 7B and E) displayed moderate, non-significant increases in abundance after prime boost, with RMs 176 and 177 being the main responders. The mean fraction of CD4 naïve T cells was significantly increased at day 35 when compared to days 24 and 28 (Fig. 7C, *P* < 0.05, two-way ANOVA, followed by Tukey’s multiple comparison test). CD4 naïve T cells continued to rebound, with the mean fraction being increased at day 42 when compared to days 7, 24, and 28, along with being significantly increased at day 64 when compared to days 35 and 42 (Fig. 7C, *P* < 0.05, two-way ANOVA, followed by Tukey’s multiple comparison test). The percentage of CD8^+^ central memory T cells was also significantly decreased at day 24 when compared to day 21 (Fig. 7D, *P* < 0.05, two-way ANOVA, followed by Tukey’s multiple comparison test). This finding suggests that the tetravalent S1 protein vaccine induces a shift toward an effector memory phenotype and away from a central memory phenotype, which may be beneficial in generating a rapid and robust response to vaccination.

**FIG 8:**
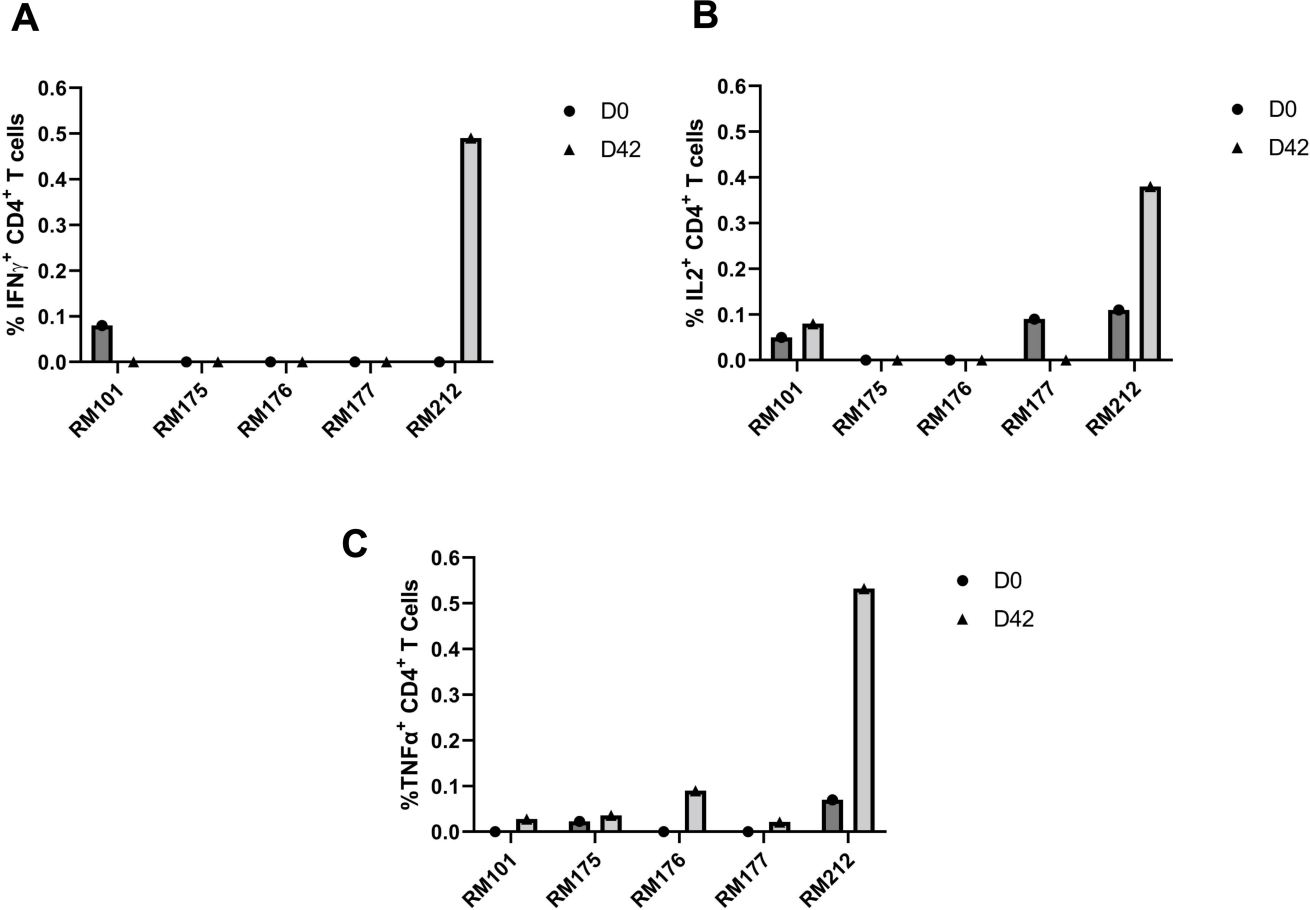
Spike-specific CD4+ T cell responses at days 0 and 42 post immunization in PBMCs. PBMCs collected prior to immunization and on day 42 post prime immunization were stimulated with PepTivator SARS-CoV-2-S1 (a pool of S1 MHC class I- and MHC class II-restricted peptides), followed by intracellular staining and flow cytometry to identify SARS-CoV-2-S1-specific T cells. (**A**) Frequencies of SARS‐CoV‐2 S1 CD4^+^ IFN‐γ^+^ T cells. Individual results for each RM are depicted. (**B**) Frequencies of SARS‐CoV‐2 S1 CD4^+^ IL-2^+^ T cells. Individual results for each RM are depicted. (**C)** Frequencies of SARS‐CoV‐2 S1 CD4^+^ TNF-α T cells. Individual results for each RM are depicted. Day 0 PBMC responses are indicated by a solid circle. Day 42 PBMC responses are indicated by a solid triangle.

Intracellular cytokine staining was performed to evaluate the spike-specific T cell responses in CD4^+^ and CD8^+^ T cells after stimulation with a spike peptide pool at days 0 and 42 postvaccination in PBMCs (Fig. 8). We tested for interferon-gamma (IFN-γ), interleukin-2 (IL-2), and tumor necrosis factor-alpha (TNF-α) cytokine staining. Only RM212 induced an IFN-γ CD4^+^ T cell response, while no such response was observed in the other four RMs (Fig. 8A). In Fig. 8B, we observed an induction of the IL-2 CD4^+^ T cell response in RM212 and, to a lesser extent, in RM101, but not in the other three RMs. Figure 8C shows an induction of TNF-α CD4^+^ T cell responses in RM212, RM176, and, to a minimal extent, RM101, RM175, and RM177. Notably, we were not able to detect a spike-specific CD8^+^ T cell response at day 0 or 42 postvaccination (data not shown). RM212 mounted a robust CD4^+^ T cell response for all three cytokines at day 42. These results suggest that there is a variable induction of cytokine responses in CD4^+^ T cells among different RMs at day 42 postvaccination.

Overall, the use of PBMCs allowed for the unique assessment of the dynamics of immune activation after vaccination. The results showed a clear increase in T cell counts and activation after boost immunization, with CD8^+^ T cell counts showing the greatest increase. The use of CD markers allowed for the differentiation of T cell subsets and their activation status, with CD8^+^ T cells expressing either CD69 or Ki-67 showing the most robust dynamics. Additionally, there was evidence of a functional spike-specific CD4^+^ T cell response in RMs at day 42 postvaccination, although in the context of no CD8^+^ T cell response. These findings highlight the potential of this vaccine candidate to induce a robust cellular immune response, which is critical for controlling viral infections.

## DISCUSSION

We evaluated the immunogenicity and efficacy of a tetravalent COVID-19 vaccine candidate based on the spike S1 protein of SARS-CoV-2 in an NHP model of controlled SIV infection. RMs infected with SIV from African green monkeys are able to control viral replication and disease progression through maintaining a healthy immune system, unlike HIV-1 in humans (44). This model shares key features of viral persistence with pathogenic HIV and SIV infections, such as rapid seeding of the viral reservoir, similar to that observed in both HIV and SIV infections; coreceptor usage and target cells infected by SIVsab, similar to both HIV and SIV infections; and a similar mechanism of viral persistence (i.e., the long decay of CM T cells and major reservoir of HIV and SIV) (43). The SIV-infected RMs in this study controlled the infection for about a year prior to SARS-CoV-2 immunization.

There were weaker bands in the western blot of the supernatant after a transient transfection with pAd/S1Alpha, pAd/S1Beta, and pAd/S1Gamma than with pAd/S1WU (Fig. 1B), which might be explained by the usage of anti-spike of SARS-CoV-2 Wuhan as a primary antibody. Indeed, no big differences were observed in the yield pre or post C-tag purification of each recombinant protein after transient transfection by sandwich ELISA with a standard of each purified rS1 protein (Fig. S1).

Our vaccine formulation induced high levels of binding antibodies against the Wuhan strain of SARS-CoV-2, as well as neutralizing antibodies against live B.1.351 (Beta) and B.1.617.2 (Delta) VOCs (Fig. 2). The sera of vaccinated RMs exhibited potent ACE2-binding inhibition capabilities against a suite of SARS-CoV-2 VOC spikes, including Omicron (BA.1) and Omicron subvariants (BA.2, BA.3, BA.1 + R246K, and BA.1 + L452R) (Fig. 3 and 4). These findings are consistent with previous studies demonstrating the immunogenicity and cross-reactivity of COVID-19 vaccines in NHP models (34, 35, 38 – 41, 49).

Importantly, the vaccine candidate also induced cellular immune responses, including T cell responses, which have been shown to play a critical role in COVID-19 immunity and protection (50 – 57). We investigated the cellular immune response to the tetravalent SARS-CoV-2 vaccine in vaccinated RMs, using a range of markers to examine T cell subsets and activation status. The results showed that the CD8^+^ T cell subset increased after the prime and after the boost, with the CD8^+^ T cell counts showing the greatest increase after prime and boost immunization compared to other cell types (Fig. 5). Mean CD20^+^ B cell counts were significantly decreased at days 42, 49, and 64 when compared to day 0, indicating a reduced number of circulating memory B cells in the peripheral blood after immunization, which may be due to the colocalization of the B cell receptor and CD20, a hallmark of terminally differentiated plasmablasts and plasma cells (58). We demonstrate that the tetravalent S1 subunit protein COVID-19 vaccine candidate induces CD4^+^ and CD8^+^ T cell activation, as indicated by increased expression of CD69, HLA-DR, CD38, and Ki-67 activation and proliferation markers on both T cell subsets (Fig. 6). However, CD4^+^ CD69^+^ T cell and CD4^+^ Ki67^+^ T cell activation was mainly seen in RM177 and to a lesser degree in other RMs.

The distribution of T cell memory subsets over time was also investigated, revealing a decrease in abundance of both CD4^+^ and CD8^+^ central memory T cells, along with CD4^+^ and CD8^+^ naïve T cells after prime and boost (Fig. 7). In contrast, CD4^+^ and CD8^+^ effector memory T cells increased in abundance after prime boost, indicating a shift toward an effector memory phenotype and away from a central memory phenotype induced by the tetravalent S1 protein vaccine (Fig. 7). Furthermore, intracellular cytokine staining was performed to evaluate the spike-specific responses of CD4^+^ and CD8^+^ T cells after stimulation with a spike peptide pool (Fig. 8). Cytokine staining for IFN-γ, IL-2, and TNF-α was tested, and a variable induction of cytokine responses by CD4^+^ T cells among different RMs at day 42 postvaccination was observed (Fig. 8). However, no spike-specific response of the CD8^+^ T cells was detected at day 0 or 42. It is possible that the spike-specific CD8^+^ T cells were present but were not detected by the intracellular staining assay, as this assay may not be sensitive enough to detect low-frequency antigen-specific CD8^+^ T cells. It is also possible that the undetectable spike-specific CD8^+^ T cell response at day 42 postvaccination was related to the time point used, which was too late after boost, as the vaccine-specific T cells had already started to wane in abundance, as shown by Arunachalam et al. (59) Altogether, our study demonstrates that the tetravalent S1 protein vaccine candidate was able to induce a robust SARS-CoV-2-specific immune response in RMs, which is promising for future development and testing of COVID-19 vaccines in humans.

The results of our study have important implications for COVID-19 vaccine development and implementation in humans. The vaccine candidate induced not only humoral immune responses but also cellular immune responses, which have been shown to be important for long-term immunity (60). The use of RMs as an animal model for studying vaccine efficacy has been widely accepted in the scientific community (33, 34, 42, 61). Here, we have used RM controllers based on the rationale that SIV controllers have a nearly healthy immune system (able to control SIV replication) (44). We also wanted to assess whether the induction of T cell activation at the effector sites would result in a burst of SIV replication. Such a boosting of SIV was reported to occur after the administration of vectorized vaccines (62). The use of NHP models has been shown to be highly informative for predicting vaccine efficacy in humans (63, 64).

The results showed that the vaccine induced both humoral and cellular immune responses against SARS-CoV-2, including neutralizing antibodies, ACE2-blocking antibodies, and T cell responses. Furthermore, the vaccine candidate was able to generate Omicron variant binding and ACE2-blocking antibodies without specifically vaccinating with Omicron, suggesting the potential for broad protection against emerging variants (65 – 69). This is particularly significant given the emergence of highly diverged SARS-CoV-2 variants, such as Omicron, which has raised concerns about vaccine efficacy and the need for updated vaccines (65, 67, 68, 70). Another significant feature of the vaccine candidate is its tetravalent composition, which targets the spike proteins of four different SARS-CoV-2 variants. This approach has the potential to provide broad protection against multiple SARS-CoV-2 variants as well as minimize the risk of immune escape and the emergence of new variants.

Protein subunit vaccines are known for their safety, ease of large-scale production, and distribution and have been used in other successful vaccine campaigns, such as the hepatitis B vaccine (63, 71 – 73). All these characteristics make protein subunit vaccines ideal candidates for worldwide vaccine equity, particularly for countries that may not have access to the more complex mRNA or viral vector vaccine platforms. Furthermore, the ability to store and transport protein subunit vaccines at a relatively low temperature (−20°C to 4°C) , compared to the ultra-low temperature required for mRNA vaccines, makes their distribution and administration easier in resource-limited settings (74, 75). The protein subunit platform is also amenable to alternative routes of administration, such as intradermal delivery, which has been shown to increase immunogenicity in other vaccine studies (20, 76 – 78). In summary, the tetravalent S1 protein subunit vaccine represents a promising vaccine candidate against SARS-CoV-2, particularly for populations that may not have access to other vaccine platforms, and could potentially be further optimized to enhance its immunogenicity.

Our study also has several limitations. The sample size was small, and we did not perform a SARS-CoV-2 virus challenge on our vaccinated RMs to fully assess vaccine efficacy (35, 59). While our results show promising immune responses to the tetravalent SARS-CoV-2 vaccine in RMs, a virus challenge would have provided further insights into the effectiveness of the vaccine in preventing infection and disease. Additionally, our study did not evaluate the durability of the antibody response generated by the vaccine over a longer period. Studies have shown that antibody responses to SARS-CoV-2 vaccines may wane over time, which highlights the importance of evaluating the longevity of vaccine-induced immunity (79 – 84). Finally, we did not assess mucosal immunity in our study, which is an important aspect of immune protection against respiratory viruses like SARS-CoV-2. Mucosal immunity may provide an additional layer of protection against infection and transmission, and future studies should investigate the mucosal immune response to the tetravalent SARS-CoV-2 vaccine (39, 85 – 88). Finally, longer intervals between prime and boost immunization could have been more immunogenic than the 3-week interval used in this study, as demonstrated in preclinical and clinical studies of SARS-CoV-2 vaccines (89 – 92). Notably, we have previously published a report describing the robust immunogenicity conferred by a booster dose of S1 protein subunit mice administered 1 year post prime immunization, suggesting that a longer interval between immunizations may also be beneficial in the context of S1-based SARS-CoV-2 vaccines (23).

The tetravalent S1 subunit protein COVID-19 vaccine candidate evaluated in this study contained SARS-CoV-2-S1 antigens from the Wuhan strain, as well as the B.1.1.7 variant, B.1.351 variant, and P.1 variant. Our study demonstrates that this vaccine candidate can induce both humoral and cellular immune responses, as evidenced by increased cell counts in both T and B cells and the production of neutralizing and cross-reactive antibodies, as well as ACE2-blocking antibodies and T cell responses. It is important to note that the RMs used in this study were infected with SIVsab and controlled the infection for a year prior to immunization. The ability of these animals to control the SIVsab infection without reactivation of the virus upon immunization while mounting immune responses to the vaccine candidate further demonstrates the potential of this vaccine candidate to provide robust protection against SARS-CoV-2, even in individuals with pre-existing conditions. Moreover, the tetravalent composition of the vaccine candidate has significant implications for COVID-19 vaccine development and implementation, with the potential to provide broad protection against multiple SARS-CoV-2 variants and to minimize the risk of immune escape and the emergence of new variants.

## MATERIALS AND METHODS

### Construction of recombinant protein-expressing vectors

The coding sequence for SARS-CoV-2-S1 amino acids 1 to 661 of full-length from BetaCoV/Wuhan/IPBCAMS-WH-05/2020 (GISAID accession ID EPI_ISL_ 403928) having a C-terminal tag known as C-tag, composed of the four amino acids, glutamic acid-proline-glutamic acid-alanine (EPEA) flanked with *Sal* I and *Not* I, was codon-optimized using the UpGene algorithm for optimal expression in mammalian cells (68) and synthesized (GenScript). The construct also contained a Kozak sequence (GCCACC) at the 5′ end. For the Alpha variant (B.1.1.7), SARS-CoV-2-S1 mutated Del69-70, Del144, N501Y, A570D, and D614G was synthesized. Also, Beta variant (B.1.351) of SARS-CoV-2-S1 (Del144, K417N, E484K, N501Y, A570D, D614G) and Gamma variant (P.1) of SARS-CoV-2-S1 (L18F, T20N, P26S, D138Y, R190S, K417T, E484K, N501Y, H655Y) were synthesized based on the above codon-optimized SARS-CoV-2-S1 Wuhan. pAd/S1WU, pAd/S1Alpha, pAd/S1Beta, and pAd/S1Gamma were then created by subcloning the four variants of codon-optimized SARS-CoV-2-S1 inserts into the shuttle vector, pAdlox (GenBank number U62024), at *Sal* I/*Not* I sites. The plasmid constructs were confirmed by DNA sequencing.

### Transient production in Expi293 cells

pAd/S1WU, pAd/S1Alpha, pAd/S1Beta, and pAd/S1Gamma were amplified and purified using the ZymoPURE II plasmid maxiprep kit (Zymo Research). For Expi293 cell transfection, we used the ExpiFectamie 293 Transfection Kit (ThermoFisher) and followed the manufacturer’s instructions. Cells were seeded at 3.0 × 10^6^ cells/mL 1 day before transfection and grown to 4.5–5.5 × 10^6^ cells/mL. Then, 1 µg of DNA and ExpiFectamine mixtures per 1 mL culture were combined and incubated for 15 min before adding 3.0  ×  10^6^ cells/mL culture. At 20 h post-transfection, enhancer mixture was added, and culture was shifted to 32°C. The supernatants were harvested 5 days post-transfection and clarified by centrifugation to remove cells, filtration through 0.8, 0.45, and 0.22 µm filters, and either subjected to further purification or stored at 4°C before purification.

### SDS-PAGE and western blot

To evaluate the expression of S1 from the plasmids, Expi293 cells were transfected with pAd/S1WU, pAd/S1Alpha, pAd/S1Beta, and pAd/S1Gamma. At 5 days after transfection, 10 µL each supernatant of Expi293 cells was subjected to SDS-PAGE and western blot as previously described (20). Briefly, after the supernatants were boiled in Laemmli sample buffer containing 2% SDS with beta-mercaptoethanol, the proteins were separated by Tris-glycine SDS-PAGE gels and transferred to a nitrocellulose membrane. After blocking for 1 h at room temperature (RT) with 5% non-fat milk in phosphate-buffered saline/Tween (PBST), rabbit anti-SARS-CoV Wuhan spike polyclonal antibody (1:3,000) (Sino Biological) was added and incubated overnight at 4 °C as primary antibody, and horseradish peroxidase (HRP)-conjugated goat anti-rabbit IgG (1:10,000) (Jackson Immunoresearch) was added and incubated at RT for 2 h as secondary antibody. After washing three times with PBST, the signals were visualized on an iBright FL 1500 Imager (ThermoFisher).

### Purification of recombinant proteins

The recombinant proteins named rS1WU, rS1Alpha, rS1Beta, and rS1Gamma were purified using a CaptureSelect C-tagXL Affinity Matrix prepacked column (ThermoFisher) and followed the manufacturer’s guidelines. Briefly, the C-tagXL column was conditioned with 10 column volumes (CV) of equilibrate/wash buffer (20 mM Tris, pH 7.4) before sample application. The supernatant was adjusted to 20 mM Tris with 200 mM Tris (pH 7.4) before being loaded onto a 5-mL prepacked column per the manufacturer’s instructions at a 5-mL/min rate. The column was then washed by alternating with 10 CV of equilibrate/wash buffer, 10 CV of strong wash buffer (20 mM Tris, 1 M NaCl, 0.05% Tween-20, pH 7.4), and 5 CV of equilibrate/wash buffer. The recombinant proteins were eluted from the column by using elution buffer (20 mM Tris, 2 M MgCl_2_, pH 7.4). The eluted solution was concentrated and desalted with preservative buffer (PBS) in an Amicon Ultra centrifugal filter device with a 50,000 molecular weight cutoff (Millipore). The concentrations of the purified recombinant proteins were determined by the BCA protein assay kit (ThermoFisher), separated by reducing SDS-PAGE, and visualized by silver staining. The rest of the proteins were aliquoted and stored at −80°C until use.

### ELISA

Sera from all rhesus macaques were collected prior to immunization and on weeks 3 and 7 after immunization. Sera was evaluated for SARS-CoV-2-S1-specific IgG using ELISA. ELISA plates were coated with 200 ng of recombinant SARS-CoV-2-S1 protein (Sino Biological) per well overnight at 4°C in carbonate coating buffer (pH 9.5) and then blocked with PBST and 2% bovine serum albumin (BSA) for 1 h. Rhesus macaque sera were inactivated at 64°C for 40 min, then diluted in PBST with 1% BSA, and incubated overnight. After the plates were washed, anti-monkey IgG-HRP (1:50,000, Sigma) was added to each well and incubated for 1 h. The plates were washed three times and developed with 3,3′5,5′-tetramethylbenzidine, and the reaction was stopped with 1 M H_2_SO_4_. Next, absorbance was determined at 450 nm using a plate reader (Molecular Devices SPECTRAmax).

### Animals and immunization

At week 0, male RMs (*n* = 5 animals per group) were bled and primed with 60 µg of tetravalent rS1 proteins from Wuhan, B.1.1.7 (Alpha), B.1.351 (Beta), and P.1 (Gamma) (15 µg of each antigen). A total volume of 300 µL of antigen was mixed with 300 µL of AddaVax adjuvant and then administered to RMs (600 µL injection volume). RMs were bled on week three and received a homologous booster of 60 µg of tetravalent rS1 proteins. RMs were bled on week 7. RMs were also bled and serially euthanized after week 9 post prime vaccination as follows: on days 0 (RM177), 1 (RM175), 6 (RM176), 8 (RM101), and 15 (RM175). PMBCs from RMs were collected and analyzed on days −1, 3, 7, 10, 14, 21, 24, 28, 31, 35, 42, 49, and 64 post prime immunization. RMs were maintained under specific pathogen-free conditions at the University of Pittsburgh.

### SARS-CoV-2 microneutralization assay

Neutralizing antibody titers against SARS-CoV-2 were defined according to the following protocol (93, 94). Briefly, 50 µL of sample from each mouse, starting from 1:10 in a twofold dilution, was added in two wells of a flat bottom tissue culture microtiter plate (COSTAR, Corning Incorporated, New York, USA), mixed with an equal volume of 100 TCID50 of a SARS-CoV-2 Wuhan, Beta, or Delta strain isolated from symptomatic patients, previously titrated, and incubated at 33°C in 5% CO_2_. All dilutions were made in Eagle’s minimum essential medium with the addition of 1% penicillin, streptomycin, glutamine, and 5 γ/mL of trypsin. After 1 h of incubation at 33°C 5% CO_2_, 3 × 10^4^ VERO E6 cells [VERO C1008 (Vero 76, clone E6, Vero E6); ATCC CRL-1586] were added to each well. After 72 h of incubation at 33°C, 5% CO_2_ wells were stained with Gram’s crystal violet solution (Merck KGaA, Damstadt, Germany) plus 5% formaldehyde at 40% mol/vol (Carlo ErbaSpA, Arese [MI], Italy) for 30 min. Microtiter plates were then washed in running water. Wells were scored to evaluate the degree of cytopathic effect (CPE) compared to the virus control. Blue staining of the wells indicated the presence of neutralizing antibodies. The neutralizing titer was the maximum dilution with a reduction of 90% of CPE. A positive titer was equal to or greater than 1:10. The GMTs of the NT_90_ endpoint titer were calculated with 4 as a negative, shown as <10. Sera from mice before vaccine administration were always included in the microneutralization (NT) assay as a negative control.

### ACE2-blocking assay

Antibodies blocking the binding of SARS-CoV-2 spike variants (Alpha [B.1.1.7], Beta [B.1.351], Gamma [P.1], Delta [B.1.617.2], Zeta [P.2], Kappa [B.1.617.1], New York [B.1.516.1], India [B.1.617 and B.1.617.3]) to ACE2 were detected with a V-PLEX SARS-CoV-2 Panel 18 (ACE2) Kit MSD according to the manufacturer’s instructions. Antibodies blocking the binding of SARS-CoV-2 spikes, including Wuhan and spikes from immune evasive variants (BA.1, BA.2, AY.4 [Delta lineage], BA.3, BA.1 + R346K mutation, BA.1 + L452R mutation, B.1.1.7 [Alpha], B.1.351 [Beta], and B.1.1640.2), to ACE2 were detected with a V-PLEX SARS-CoV-2 Panel 25 (ACE2) Kit MSD according to the manufacturer’s instructions. Serum samples were diluted (1:10 and 1:100). The assay plate was blocked for 30 min and washed. Serum samples were diluted (1:10 for P18, 1:10 and 1:100 for P25), and 25 µL was transferred to each well. The plate was then incubated at room temperature for 60 min with shaking at 700 rpm, followed by the addition of SULFO-TAG-conjugated ACE2, and continued incubation with shaking for 60 min. The plate was washed; 150 µL MSD GOLD Read Buffer B was added to each well; and the plate was read using the QuickPlex SQ 120 Imager. Electrochemiluminescent values were generated for each sample. Results were calculated as % inhibition compared to the negative control for the ACE2 inhibition assay, and % inhibition is calculated as follows: % neutralization = 100 × [1 − (sample signal / negative control signal)].

### Flow cytometry

Absolute counts of immune cells in whole blood and immunophenotyping of circulating immune cells were performed by flow cytometry. First, 50 µL of whole blood was added to a TruCount tube (BD Biosciences) containing an antibody mix, allowing to precisely quantify CD45^+^ cell counts in blood, as well as CD4^+^ and CD8^+^ T cells and CD20^+^ B cells. Whole peripheral blood was stained with fluorescently labeled antibodies (all purchased from BD Bioscience, San Jose, CA, USA, unless noted otherwise) as follows: CD3 (clone SP34-2, V450), CD4 (clone L200, APC), CD8 (clone RPA-T8, PE-CF594), CD28 (clone CD28.2, PE-Cy7), CD38 (clone AT-1, FITC) (Stemcell), CD45 (clone D058-1283, PerCP), CD69 (clone FN50, APC-H7), CD95 (clone DX2, FITC), HLA-DR (clone L243, PE-Cy7), and Ki-67 (clone P56, PE). For intracellular staining, cells were fixed and permeabilized with 1× BD Fix/Perm before being stained for Ki-67. Flow cytometry acquisitions were performed on an LSRFortessa flow cytometer (BD Biosciences), and flow data were analyzed using FlowJo v10.8.0 (TreeStar, Ashland, OR, USA).

### Spike-specific intracellular staining

Antigen-specific T cell responses in the PBMCs of RMs immunized as described above were analyzed after immunization by flow cytometry, adhering to the recently published guidelines (21, 95). PBMCs collected prior to immunization and on day 42 post prime immunization were stimulated with PepTivator SARS-CoV-2-S1 (a pool of S1 MHC class I- and MHC class II-restricted peptides) overnight in the presence of protein transport inhibitors (Golgi Stop) for the last 4 h. Unstimulated cells were used as negative controls. Phorbol myristate acetate and ionomycin-stimulated cells served as positive controls. Cells were washed with FACS buffer (PBS, 2% fetal calf serum [FCS]), incubated with Fc Block (BD Biosciences, 553142) for 5 min at 4°C, and stained with surface marker antibody (Ab) stain for 20 min at 4°C. Surface Abs were used as follows: CD3-V450 (SP34-2, V450; BD Biosciences), CD4-APC (L200, APC; BD Biosciences), and CD8ab-PE-CF594 (RPA-T8, PE-CF594; BD Biosciences). For dead cell exclusion, cells were stained with Zombie NIR Fixable Viability dye (BioLegend) for 10 min at 4°C and washed in FACS buffer. Intracellular cytokine staining was performed on surface Ab-stained cells by first fixing and permeabilizing cells using the FoxP3 Transcription Factor Staining Buffer kit (eBioscience, 00-5523-00) following the manufacturer’s instructions. Intracellular staining with IFN-γ-FITC (4S.B3, FITC; BD Biosciences), IL-2-PE (MQ1-17H12, PE; BD Biosciences), and TNFa-AF700 (Mab11, AF700; BD Biosciences). Samples were run on an Aurora (Cytek) flow cytometer, and flow data were analyzed using FlowJo v10.8.0 (TreeStar, Ashland, OR, USA).

### Statistical analysis

Statistical analyses were performed using GraphPad Prism v9 (San Diego, CA, USA). Significant differences are indicated by *P < 0.05. Comparisons with nonsignificant differences are not indicated.

## SUPPLEMENTAL MATERIAL

Supplemental figures mbio.02070-23-s0001.pdf Fig. S1 and S2.

Supplemental legends mbio.02070-23-s0002.docx Legends for Fig. S1 and S2.

ASM does not own the copyrights to Supplemental Material that may be linked to, or accessed through, an article. The authors have granted ASM a non-exclusive, world-wide license to publish the Supplemental Material files. Please contact the corresponding author directly for reuse.
