# Supplementary figures and images for: Tetravalent SARS-CoV-2 S1 subunit protein vaccination elicits robust humoral and cellular immune responses in SIV-infected rhesus macaque controllers
Source: mBio. 2023 Oct 13;14(5):e02070-23. doi: 10.1128/mbio.02070-23 (PMC10653869; doi:10.1128/mbio.02070-23)

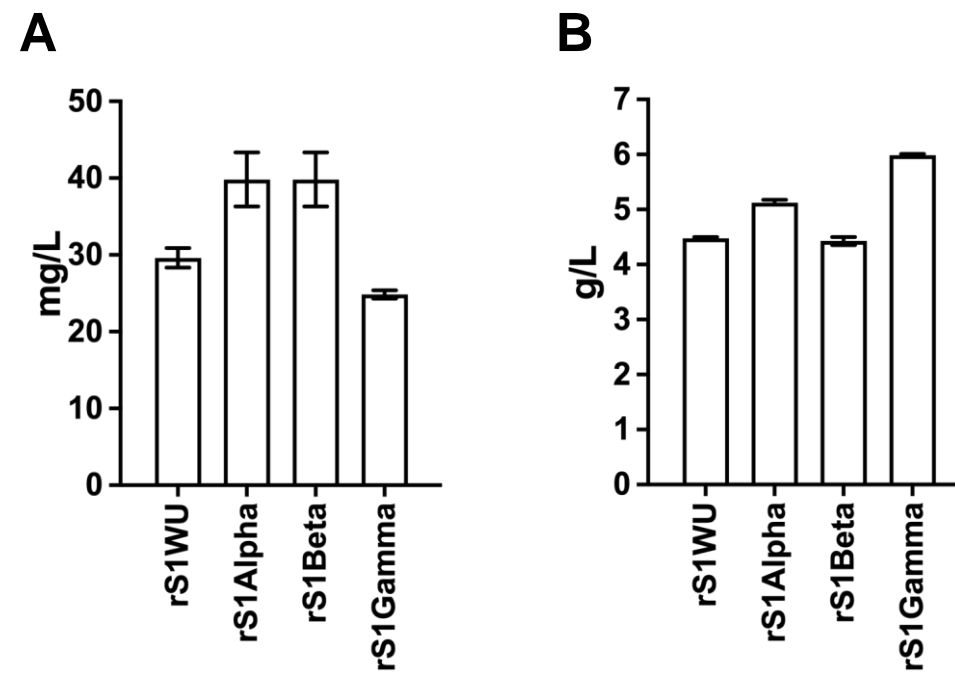

Supplementary Figure 1

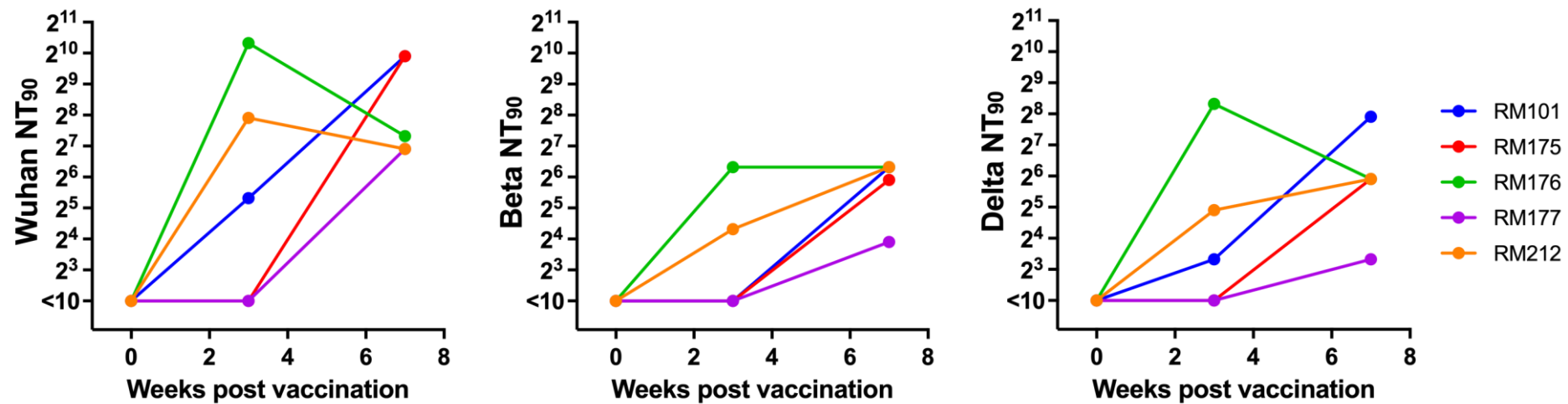

Supplementary Figure 2

Supplement: Supplemental figures — Fig. S1 and S2. [file mbio.02070-23-s0001.pdf]
