# Supplementary material for: Tetravalent SARS-CoV-2 S1 subunit protein vaccination elicits robust humoral and cellular immune responses in SIV-infected rhesus macaque controllers
Source: mBio. 2023 Oct 13;14(5):e02070-23. doi: 10.1128/mbio.02070-23 (PMC10653869; doi:10.1128/mbio.02070-23)
Supplement: Supplemental legends — Legends for Fig. S1 and S2. [file mbio.02070-23-s0002.docx]

**Supplementary Figure 1. Yield pre and post C-tag purification of each recombinant proteins after transient transfection.** To evaluate the expression of rS1WU, rS1Alpha, rS1Beta, and rS1Gamma recombinant proteins, ELISA plates were coated with chimeric MAb 40150-D003 (1:750, Sino Biological) overnight at 4°C. **A.** The supernatants of Expi293^TM^ cells transfected with pAd/S1WU, pAd/S1Alpha, pAd/S1Beta, and pAd/S1Gammawas, respectively, diluted 1:40 or **B.** purified each protein by a CaptureSelect^TM^ C-tagXL Affinity Matrix prepacked column diluted 1:1000 in PBS-T with 1% BSA and along with each purified rS1 proteins for a standard curve were incubated overnight at 4°C. After the plates were washed, chimeric MAb 40150-D001 HRP conjugated secondary antibody (1:10000, Sino Biological) was added to each well. After the development with reagent, the reaction was determined using an ELISA reader (Molecular Devices SPECTRAmax) in same as described in materials and methods.

**Supplementary Figure 2.** Neutralizing antibodies at week 0, 3, and 7 using a microneutralization assay (NT_90_) were showed in each RM with SARS-CoV-2 Wuhan, Beta, and Delta variants.
